# The GRADE Evidence to Decision (EtD) framework for health system and public health decisions

**DOI:** 10.1186/s12961-018-0320-2

**Published:** 2018-05-29

**Authors:** Jenny Moberg, Andrew D. Oxman, Sarah Rosenbaum, Holger J. Schünemann, Gordon Guyatt, Signe Flottorp, Claire Glenton, Simon Lewin, Angela Morelli, Gabriel Rada, Pablo Alonso-Coello, for the GRADE Working Group, Jenny Moberg, Andrew Oxman, Pablo Alonso Coello, Holger Schünemann, Gordon Guyatt, Sarah Rosenbaum, Angela Morelli, Elie Akl, Claire Glenton, Metin Gulmezoglu, Signe Flottorp, Simon Lewin, Reem A. Mustafa, Gabriel Rada, Jasvinder Singh, Erik von Elm, Josh Vogel, Joseph Watine

**Affiliations:** 1 Norwegian Institute of Public Health, PO Box 4404, Nydalen, N-0403 Oslo, Norway; 2 Department of Medicine, McMaster University, Hamilton, Canada; 3 Department of Clinical Epidemiology and Biostatistics, McMaster University, Hamilton, Canada; 4 Health Systems Research Unit, South African Medical Research Council, Cape Town, South Africa; 5 Evidence-Based Health Care Program, Pontificia Universidad Católica de Chile, Santiago, Chile; 6 Iberoamerican Cochrane Centre, CIBERESP-IIB Sant Pau, Barcelona, Spain

**Keywords:** Decision-making, Health systems, Public health, GRADE, Evidence to decision, Recommendations, Methodology

## Abstract

**Objective:**

To describe a framework for people making and using evidence-informed health system and public health recommendations and decisions.

**Background:**

We developed the GRADE Evidence to Decision (EtD) framework for health system and public health decisions as part of the DECIDE project, in which we simultaneously developed frameworks for these and other types of healthcare decisions, including clinical recommendations, coverage decisions and decisions about diagnostic tests.

**Developing the framework:**

Building on GRADE EtD tables, we used an iterative approach, including brainstorming, consultation of the literature and with stakeholders, and an international survey of policy-makers. We applied the framework to diverse examples, conducted workshops and user testing with health system and public health guideline developers and policy-makers, and observed and tested its use in real-life guideline panels.

**Findings:**

All the GRADE EtD frameworks share the same basic structure, including sections for formulating the question, making an assessment and drawing conclusions. Criteria listed in the assessment section of the health system and public health framework cover the important factors for making these types of decisions; in addition to the effects and economic impact of an option, the priority of the problem, the impact of the option on equity, and its acceptability and feasibility are important considerations that can inform both whether and how to implement an option. Because health system and public health interventions are often complex, detailed implementation considerations should be made when making a decision. The certainty of the evidence is often low or very low, but decision-makers must still act. Monitoring and evaluation are therefore often important considerations for these types of decisions.

We illustrate the different components of the EtD framework for health system and public health decisions by presenting their application in a framework adapted from a real-life guideline.

**Discussion:**

This framework provides a structured and transparent approach to support policy-making informed by the best available research evidence, while making the basis for decisions accessible to those whom they will affect. The health system and public health EtD framework can also be used to facilitate dissemination of recommendations and enable decision-makers to adopt, and adapt, recommendations or decisions.

**Electronic supplementary material:**

The online version of this article (10.1186/s12961-018-0320-2) contains supplementary material, which is available to authorized users.

## Background

Policy-makers or managers, often in groups or individually whilst advised by a group, have the mandate to set priorities and make health system and public health decisions on behalf of a population. Health systems require decisions about how services are delivered, financed and governed, and about implementation strategies [1–4]. Public (population) health decisions that are made on behalf of a population include decisions about whether to offer organised screening and other public health programmes, environmental and occupational health policies, injury prevention policies, and nutrition and food safety policies. Such decisions can be made at an international level (e.g. WHO guidelines), a national level (e.g. deciding on national regulations for physicians and other health professionals) or a local level (e.g. deciding whether to care for acute stroke patients in a specialised stroke unit or a general medical ward).

All healthcare decision-making is complex. Decision-makers may not have clear criteria, may sometimes neglect important criteria, give inappropriate importance to certain criteria, or may not use the best available evidence to inform their judgments. Structured and transparent systems for decision-making can help to ensure that all important criteria are considered and that the best available research evidence is used.

In this article, we present, and illustrate with an example, the GRADE Evidence to Decision (EtD) framework for people who make or use health system and public health recommendations and decisions. We will use the term ‘health system and public health decisions’ to indicate both recommendations and decisions, and we will refer to the group of people making a decision or advising decision-makers as the ‘panel’.

## GRADE EtD frameworks

Building on the GRADE approach to making judgments about the strength of recommendations and EtD tables [5–8], the DECIDE project [9] developed EtD frameworks for different types of healthcare decisions, including clinical recommendations, coverage decisions, and health system or public health recommendations and decisions. This is described in detail elsewhere [10–14].

**Fig. 1:**
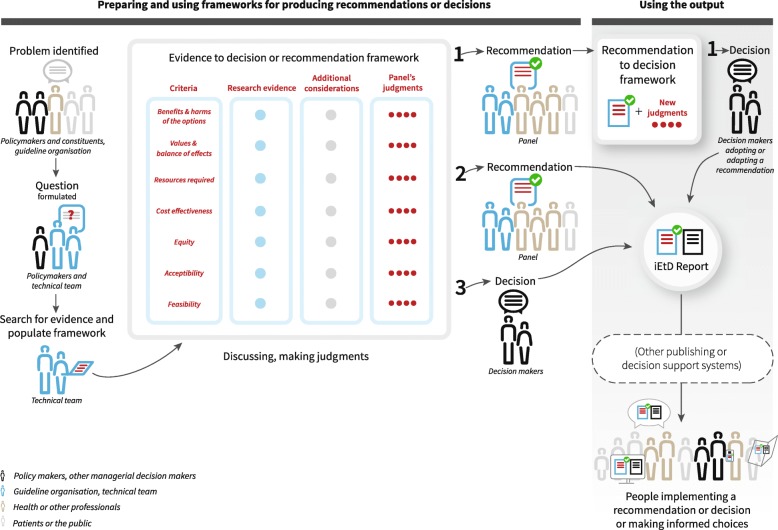
Evidence to Decision (EtD) conceptual map workflow

Figure 1 shows how EtD frameworks can fit into practical decision-making processes, who is involved, and who the target audience for the framework is. For any healthcare decision, policy-makers and their constituents identify and prioritise problems, then work with a technical team to formulate a question. The technical team searches for and summarises the evidence and populates the EtD framework. The framework is then used by a panel to facilitate and document their decision-making, and later by people who will either use or be affected by a decision, including policy-makers, health professionals, patients and the public. Additional file 1 is a glossary of terminology used in EtD frameworks for all types of healthcare decisions.

### The purpose of the frameworks

The main purpose of EtD frameworks is to help panels use evidence in a systematic and transparent way to inform decisions [10]. EtD frameworks support panels by informing panel members about the relative pros and cons of the interventions or options being considered; ensuring that panel members consider all the important factors (criteria) for making a decision; providing panel members with a concise summary of the best available evidence about each criterion to inform their judgments; helping panels structure and document discussion; and helping panels identify reasons for disagreements, making the process and the basis for decisions transparent.

EtD frameworks support users of recommendations and those affected by decisions by assisting them to understand the judgments made by the panel and the evidence supporting those judgments; helping policy-makers and managers consider whether recommendations can and should be implemented in their own settings; and facilitating adoption of decisions and adaptation of recommendations to specific contexts.

## Developing the GRADE EtD framework for health system and public health decisions

We aimed for consistency across the EtD frameworks for different types of decisions; most of the criteria are similar across the frameworks, as can be seen in Table 1, which shows the criteria for five types of decisions. However, different types of decisions and varying perspectives require different considerations. Consequently, the specific criteria for health system and public health decisions differ in some ways from the criteria for clinical recommendations, coverage decisions, and recommendations and decisions about tests.

**Table 1:** Criteria for Evidence to Decision (EtD) frameworks for five different types of decisions

	Clinical recommendations – individual perspective	Clinical recommendations – population perspective	Coverage decisions	Health system and public health recommendations	Diagnostic, screening and other tests (clinical and public health recommendations – individual and population perspective)
Priority of the problem	Is the problem a priority?				
Test accuracy	Not applicable				How accurate is the test?
Benefits & harms	How substantial are the desirable anticipated effects?				
	How substantial are the undesirable anticipated effects?				
Certainty of the evidence	What is the overall certainty of the evidence of effects?				What is the certainty of the evidence of test accuracy? What is the certainty of the evidence for any critical or important direct benefits, adverse effects or burden of the test? What is the certainty of the evidence of effects of the management that is guided by the test results? How certain is the link between test results and management decisions? What is the overall certainty of the evidence of effects of the test?
Outcome importance	Is there important uncertainty about or variability in how much people value the main outcomes?	Is there important uncertainty about or variability in how much people value the main outcomes?	Is there important uncertainty about how much people value the main outcomes?	Is there important uncertainty about or variability in how much people value the main outcomes?	Is there important uncertainty about or variability in how much people value the main outcomes, including adverse effects and burden of the test and downstream outcomes of clinical management that is guided by the test results?
Balance	Does the balance between desirable and undesirable effects favour the intervention or the comparison?				Does the balance between desirable and undesirable effects favour the test or the comparison?
Resource use		How large are the resource requirements (costs)?			
		What is the certainty of the evidence of resource requirements (costs)?			
	Does the cost-effectiveness of the intervention (the out-of-pocket cost relative to the net benefits) favour the intervention or the comparison?	Does the cost-effectiveness of the intervention favour the intervention or the comparison?	Does the cost-effectiveness of the intervention favour the intervention or the comparison?	Does the cost-effectiveness of the intervention favour the option or the comparison?	Does the cost-effectiveness of the test favour the test or the comparison?
Equity		What would be the impact on health equity?			
Acceptability	Is the intervention acceptable to patients, their caregivers and healthcare providers?	Is the intervention acceptable to key stakeholders?	Is the intervention acceptable to key stakeholders?	Is the option acceptable to key stakeholders?	Is the test acceptable to key stakeholders?
Feasibility	Is the intervention feasible for patients, their caregivers and healthcare providers?	Is the intervention feasible to implement?	Is the intervention feasible to implement?	Is the option feasible to implement?	Is the test feasible to implement?

We developed the EtD framework for health system and public health decisions using an iterative approach, as described in a previous publication [9]. Additional input came from experience with evidence-based policy briefs [15, 16]. We obtained feedback from an international stakeholder group including health system and public health researchers and policy-makers, and undertook an international survey of policy-makers’ perceptions regarding the criteria in the framework and of how best to summarise and present evidence to support health systems [17]. We applied the framework to a diverse set of examples [18–23], conducted workshops with guideline developers and policy-makers, and observed guideline panels using the framework. Finally, we undertook user testing [24] with members of guideline panels and with policy-makers.

Below we describe the EtD framework for health system and public health decisions, and highlight its differences from the EtD frameworks for other types of decisions. Similarities and differences between the EtD framework for health system and public health decisions and other EtD frameworks are summarised in Table 2. To illustrate the application of each component of the health system and public health EtD framework, we present an example of its use, namely a recommendation regarding women’s groups using participatory learning and action (PLA) cycles to achieve a positive pregnancy experience. We prepared this framework, presented fully in Additional file 2, based on two WHO guidelines, one on antenatal care for a positive pregnancy experience [18] and one on community mobilisation through facilitated PLA cycles with women’s groups for maternal and newborn health [19], in which the EtD for health system and public health decisions was used. We modified the EtD frameworks used in these two guidelines to reconcile differences between them and to clarify the use of the framework in relation to some of its specific components.

**Table 2:** Similarities and differences between the Evidence to Decision (EtD) framework for health system and public health decisions and other types of decisions

Similarities	
Structure of the EtD framework	All EtD frameworks include three sections: the question, an assessment and conclusions
Question	All EtD frameworks include question details, which include the setting and perspective that is taken, subgroups that are important to consider, and background information. They all also include a summary of declarations of interest for each panel member and how they were managed
Assessment	All EtD frameworks include criteria and, for each criterion, a judgment, research evidence to inform the judgment, additional considerations and detailed judgments. All of the EtD frameworks include criteria for the priority of the problem, how substantial the benefits and harms are, the certainty of the evidence, how much people value the main outcomes, the balance between the desirable and undesirable effects, cost-effectiveness, acceptability and feasibility
Conclusions	All of the EtD frameworks include a summary of judgments, decision options (types of recommendations or decisions), the decision, a justification for the decision, subgroup considerations (or, for coverage decisions, ‘restrictions’), implementation considerations, and monitoring and evaluation considerations
Multiple options	For each type of decision there are templates to accommodate decisions when there are more than two options (Box 1)
Differences	
Nature of the decision	Health system and public health decisions (and coverage decisions) are made by policy-makers or managers on behalf of a population, whereas clinical decisions are typically made by individuals (health professionals or patients)
Question details	Other EtD frameworks, for the most part, use ‘PICO’– patients, intervention, comparison and outcomes. Health system and public health decisions typically begin with a problem and consider options for addressing the problem, so ‘POCO’ is used – problem, option, comparison and outcomes
Priority of the problem	The number of people affected is important to consider when making a judgment about the priority of a health system and public health problem, whereas it is not directly relevant for other types of decisions. The number of people affected by a problem can influence a clinical recommendation (from a population perspective) or coverage decision because of the impact on resource requirements (the more people affected, the greater the cost). However, this is addressed directly by another criterion: How large are the resource requirements (costs)? A problem is not more or less important to the people with the problem because of the number of people affected and most people would not consider a severe problem to be more or less important to treat depending on the number of people affected
Benefits and harms	Policy-makers and managers often must make health system and public health decisions when the certainty of the evidence is low or very low, and they also need to consider indirect as well as direct effects; this is not unique for health system and public health decisions, but is more characteristic and important than for other types of decisions
Resources	Because resources are limited, policy-makers and managers making health system and public health decisions must consider the resource implications of implementing alternative options; this also is not unique for health system and public health decisions, but is more characteristic and important than for other types of decisions
Equity	Consideration of impacts on equity is more important for health system and public health options than for clinical recommendations, although it also is sometimes important for clinical recommendations.
Acceptability	Consideration of acceptability is more important for health system and public health options than for clinical recommendations, and acceptability typically needs to be considered for multiple stakeholders, more so than for clinical recommendations and coverage decisions
Feasibility	Consideration of feasibility is more important for health system and public health options than for clinical recommendations
Decisions	EtD frameworks for clinical decisions are designed to be used by panels to make recommendations, although they can be used as the basis for decision-support tools for individual patients and clinicians. EtD frameworks for health system and public health decisions (and for coverage decisions) can be used directly for decisions or for recommendations (Box 1)
Monitoring and evaluation	Because there is often important uncertainty about the effects of health system and public health interventions, monitoring and evaluation considerations are a key element of these EtD frameworks

## Description of the health system and public health EtD framework

The EtD framework includes three main sections that reflect key steps in going from evidence to a decision, namely formulating the question, making an evidence-informed assessment and drawing conclusions. The EtD framework for health system and public health decisions shares the same basic structure as the EtD frameworks for other types of decisions [10]. Additionally, as for other types of decisions, the framework accommodates both recommendations and decisions with either two options or multiple options (Box 1).

### Formulating the question

Panels, sometimes together with a technical team, formulate questions by defining the problem they are addressing (e.g. perinatal and maternal mortality, morbidity and pregnancy experience) and considering options to address it (e.g. women’s groups using PLA cycles). In the framework and in this article, we use the term ‘options’ to describe the interventions or actions available to address a problem. The question also includes the comparison option, the main outcomes, the setting and the perspective from which the decision is being made. Given that their decisions will affect groups of people, or a whole population, health system and public health decision-makers take a population perspective. The specific type of population perspective taken depends on the nature of the decision. For example, a decision about an environmental health policy might be made from a societal perspective (incorporating all important outcomes and all costs and savings, regardless of who benefits or pays). In the participatory women’s groups example, the panel took a population perspective, and considered costs and savings within the healthcare system.

In the example framework in Additional file 2, the team developing this WHO guideline formulated the ‘problem’ positively as a goal, that of a positive pregnancy experience. A positive pregnancy experience was defined as maintaining physical and sociocultural normality, maintaining a healthy pregnancy for mother and baby (including preventing or treating risks, illness and death), having an effective transition to positive labour and birth, and achieving positive motherhood (including maternal self-esteem, competence and autonomy).

The option considered in the framework in Additional file 2 was women’s groups using PLA cycles and the comparison was usual care. Women’s groups using PLA cycles are facilitated by trained facilitators, with the aim of identifying, prioritising and addressing problems women face around pregnancy, childbirth and after birth, and empowering women to seek care and choose healthy pregnancy and newborn care behaviours. Meetings are usually held on a monthly basis and activities are differentially prioritised according to the local context and conditions.

The main outcomes considered were attending four or more antenatal care visits, attending one or more antenatal care visit, delivery in a health facility, perinatal deaths and maternal deaths. The decision setting was a global recommendation, and the perspective was that of ministries of health.

It is often important to consider subgroups or contexts for which it may be appropriate to make separate judgments, and potentially different decisions, particularly when making global recommendations. These should be identified when the question is formulated to ensure that panels consider relevant evidence and their judgments for different subgroups or contexts when making an assessment. For example, although we have not specified any subgroups in the framework in Additional file 2, the WHO panel specifically considered women in rural settings with low access to health services in making its recommendation.

Intellectual and financial conflicts of interest are common and can affect judgments and recommendations or decisions [25–29]. Guideline developers and organisations responsible for healthcare decisions should consider conflicts of interest when a panel is established [29]. In addition, because potential conflicts of interest can vary across questions, panels should consider and report them when formulating each question. They should also specify actions to address these, which can range from simply declaring a conflict of interest to excluding panel members from discussions of specific questions or an entire guideline [27–29]. In the participatory women’s groups example, the panel reported that all panellists declared either non-important minor or no conflicts of interest (Additional file 2).

### Making an evidence-informed assessment

The EtD framework lists explicit criteria that are important to consider when making a decision. The EtD framework criteria for different types of healthcare decisions are listed in Table 1. For each criterion, the technical team enters a summary of the research evidence and any information for additional consideration by the panel, with links to more detailed information. The panel assesses the research evidence and additional considerations presented, and makes an informed judgment about the options. Research evidence refers to information derived from studies that used systematic and explicit methods. Additional considerations include other evidence, such as routinely collected data, and assumptions and logic used to make a judgment. Panels may make different judgments for one or more subgroups in relation to some or all of the criteria. When relevant, they may also report additional details, such as dissenting views of panel members or the results of voting on judgments for which there was disagreement.

The criteria in the EtD framework for health system and public health decisions include questions about whether the problem is a priority, the magnitude of the desirable and undesirable effects, the certainty of the evidence, consideration of how people who are directly affected value the main outcomes, the balance between desirable and undesirable effects, resource use and cost-effectiveness, impacts on equity, and the acceptability and feasibility of the option. Table 3 shows other criteria that we have incorporated in the framework as detailed judgments, which some organisations might want to consider as separate criteria. For example, a Swedish group that assessed the applicability of an earlier version of the EtD framework for public health decisions concluded that two criteria which we have included as detailed judgments should be added as criteria [23]. The first, individual autonomy, is a detailed judgment for the criterion for acceptability, and the second, sustainability, is a detailed judgment for feasibility (Table 3).

**Table 3:** Detailed judgments in Evidence to Decision (EtD) frameworks

Criterion	Detailed judgments
Is the problem a priority?^a^	• Are the consequences of the problem serious (i.e. severe or important in terms of the potential benefits or savings)? • Is the problem urgent? [not relevant for coverage decisions] • Is it a recognised priority (e.g. based on a political or policy decision)? [Not relevant when an individual patient perspective is taken]
How substantial are the desirable anticipated effects?	• Judgments for each outcome for which there is a desirable effect
How substantial are the undesirable anticipated effects?	• Judgments for each outcome for which there is an undesirable effect
What is the overall certainty of the evidence of effects?	• See GRADE guidance regarding detailed judgments about the quality of evidence or certainty in estimates of effects
Is there important uncertainty about or variability in how much people value the main outcomes?	• Is there important uncertainty about how much people value each of the main outcomes? • Is there important variability in how much people value each of the main outcomes? [not relevant for coverage decisions]
Do the desirable effects outweigh the undesirable effects?	• Judgments regarding each of the four preceding criteria • To what extent do the following considerations influence the balance between the desirable and undesirable effects: - How much less people value outcomes that are in the future compared to outcomes that occur now (their discount rates) - People’s attitudes towards undesirable effects (how risk averse they are) - People’s attitudes towards desirable effects (how risk seeking they are)
How large are the resource requirements?^a^	• How large is the difference in each item of resource use for which fewer resources are required? • How large is the difference in each item of resource use for which more resources are required?
What is the certainty of the evidence of resource requirements?^b^	• Have all-important items of resource use that may differ between the options being considered been identified? • How certain is the evidence of differences in resource use between the options being considered? (see GRADE guidance regarding detailed judgments about the quality of evidence or certainty in estimates) • How certain is the cost of the items of resource use that differ between the options being considered? • Is there important variability in the cost of the items of resource use that differ between the options being considered?
Are the net benefits worth the incremental cost?^a^	• Judgments regarding each of the six preceding criteria • Is the cost-effectiveness ratio sensitive to one-way sensitivity analyses? • Is the cost-effectiveness ratio sensitive to multi-variable sensitivity analyses? • Is the economic evaluation on which the cost-effectiveness estimate is based reliable? • Is the economic evaluation on which the cost-effectiveness estimate is based applicable to the setting(s) of interest?
What would be the impact on health equity?^a,b^	• Are there groups or settings that might be disadvantaged in relation to the problem or options that are considered? • Are there plausible reasons for anticipating differences in the relative effectiveness of the option for disadvantaged groups or settings? • Are there different baseline conditions across groups or settings that affect the absolute effectiveness of the intervention or the importance of the problem for disadvantaged groups or settings? • Are there important considerations that should be made when implementing the intervention in order to ensure that inequities are reduced, if possible, and that they are not increased?
Is the intervention acceptable to key stakeholders?^a^	• Are there key stakeholders that would not accept the distribution of the benefits, harms and costs? • Are there key stakeholders that would not accept the costs or undesirable effects in the short term for desirable effects (benefits) in the future? • Are there key stakeholders that would not agree with the values attached to the desirable or undesirable effects (because of how they might be affected personally or because of their perceptions of the relative importance of the effects for others)? • Would the intervention adversely affect people’s autonomy? • Are there key stakeholders that would disapprove of the intervention morally, for reasons other than its effects on people’s autonomy (e.g. in relation to ethical principles such as no maleficence, beneficence or justice)?
Is the intervention feasible to implement?^a^	*For decisions other than coverage decisions:* • Is the intervention or option sustainable? • Are there important barriers that are likely to limit the feasibility of implementing the intervention (option) or require consideration when implementing it? *For coverage decisions:* • Is coverage of the intervention sustainable? • Is it feasible to ensure appropriate use for approved indications? • Is inappropriate use (indications that are not approved) an important concern? • Is there capacity to meet increased demand if covered? • Are there important legal or bureaucratic or ethical constraints that make it difficult or impossible to cover the intervention?

^a^The certainty of the evidence could be considered as a detailed judgment for these criteria

^b^These criteria are not included when an individual patient perspective is taken

#### The problem

Healthcare decisions require setting priorities on how best to use limited resources. In considering whether the problem being addressed in a health system and public health decision is a priority, the number of people affected is important to consider, in addition to the severity, urgency and consequences of the problem, and whether it is a recognised priority (e.g. based on a national health plan or international health targets such as the UN Sustainable Development Goals). The more people that are affected, the more likely it is that doing something to address the problem should be a priority. For example, governments might prioritise problems that represent a larger burden of disease for their population or that place a larger burden on the healthcare system by making an explicit comparison with other health problems of mortality figures and disability adjusted life years.

In the example in Additional file 2, the problem is achieving a positive pregnancy experience. Approximately 303,000 women and adolescent girls died as a result of pregnancy and childbirth-related complications, and approximately 2.6 million babies were stillborn in 2015 [30, 31]. Almost two thirds of the global maternal and neonatal disease burden could be alleviated through optimal adaptation and uptake of existing research findings [32]. In addition, reducing maternal and neonatal mortality are targets of Sustainable Development Goal 3 (ensuring health and well-being for all). Based in part on this evidence, the panel concluded that achieving a positive pregnancy experience is a priority. This judgment applied to all of the panel’s recommendations in the guideline and did not need to be repeated for each one.

#### Benefits and harms

Panels should consider the evidence about the benefits and harms of the options and how certain that evidence is. They also need to consider how much the people affected directly by the decision value the benefits and harms, whether there is important uncertainty about this, and whether there is important variability in how much people value the benefits and harms [11]. They must then consider all these criteria together to make a judgment about the balance between the desirable and undesirable effects of the option.

The certainty of the evidence for health system and public health interventions is often low or very low [1–4]. Nonetheless, policy-makers and managers must still make decisions. Panels need to consider indirect as well as direct effects of options; for example, herd immunity may be an important consideration for immunisation programmes in addition to the direct benefits and harms experienced by the people who are vaccinated. How much the people affected by the decision value the outcomes is particularly important when potential harms and benefits are closely balanced. The more uncertainty or variability there is about how much those affected value the main outcomes, the less likely a panel is to make a strong recommendation for an option. There is often a paucity of evidence about how much people value important outcomes. When this is the case, panels should state the basis for their judgments and any assumptions that they have made.

In the example in Additional file 2, the panel judged that the desirable effects of participatory women’s groups are moderate, but uncertain, the undesirable effects are trivial, and the overall certainty of the effects is low. The panel was not presented any evidence of how much women value the main outcomes that were considered. However, women’s groups very likely increase communication and social support, which women value [33]. Given that no adverse effects of women’s groups were identified, there was no important uncertainty or variability in how much women value the main outcomes. Based on these judgments, the panel determined that the balance of the desirable and undesirable effects probably favours the option of women’s groups using PLA cycles.

#### Resource use and cost-effectiveness

Because resources are limited, policy-makers and managers making health system and public health decisions must consider the resource implications of implementing alternative options, in addition to the extent to which an option is cost-effective. Typically, there are uncertainties regarding the inputs that are required, the effects, the economic consequences of those effects, and the resources consumed or saved due to implementation of an option. There may be limited or no available economic analyses, nor resources available to conduct one [34, 35]. As with clinical recommendations and coverage decisions, the greater the budget impact and the more uncertainty there is about the resources required and cost effectiveness, the less likely a panel is to recommend or decide to implement a health system or public health option [11, 13]. Conversely, the greater the savings, the more appealing an option becomes.

For the example in Additional file 2, amongst other costs, facilitators’ time, training and supervision must be considered. These costs are difficult to estimate and may vary widely across settings [36]. The panel’s judgment was that there are moderate costs associated with implementing women’s groups using PLA cycles, but that the certainty of this evidence was low.

On the other hand, a systematic review of the cost-effectiveness of strategies to improve the utilisation and provision of maternal and newborn healthcare in low- and lower middle-income countries reported that there was high certainty evidence for the cost-effectiveness of women’s groups using PLA cycles [36, 37]. The panel’s judgment was that, despite wide variation in the incremental cost per neonatal death averted in the included trials, the available cost-effectiveness evidence probably favours the option of women’s groups using PLA cycles.

#### Equity

The impacts on equity of health system and public health options are important because these decisions are always taken from a population perspective. Decision-makers can address potential impacts on equity by considering the possible differential effects of options on disadvantaged populations, and in relation to characteristics that are likely to be associated with disadvantage [38, 39], including economic status, employment or occupation, education, place of residence, sex and ethnicity. Decision-makers also need to consider who will bear the costs (or benefit from the savings) of options, and the impacts that this will have on equity [38].

In the example in Additional file 2, there is evidence that the effect of participatory women’s groups on neonatal mortality rates was greatest among the most socioeconomically marginalised [40, 41]. Participatory women’s groups are one way of facilitating dialogue with, and empowering, disadvantaged populations to engage in efforts to improve health and to strengthen broader community support. Based on this evidence, the panel’s judgment was that women’s groups using PLA cycles probably increase equity.

#### Acceptability

Decision-makers should consider the acceptability of options – and of decisions of whether or not to implement them – to all the key stakeholders. For health system and public health decisions, key stakeholders may include those affected by the problem and the option, public officials and politicians, healthcare managers, the general public, health workers and their unions, and special interest groups [42]. The acceptability of an option may depend on evidence presented for some of the preceding criteria, such as the distribution and timing of harms, benefits and costs, and how much different stakeholders value the harms and benefits.

An option might be more or less acceptable to some stakeholders depending on the distribution of the impacts and demands of an option across the people affected, including the burden. For example, shifting tasks from one health worker cadre to another (e.g. from midwives to lay health workers) might increase both groups’ job satisfaction, but might also increase the workload for one of these groups, making that option more, or less, acceptable to them [43].

The timing of the impacts and demands of an option might also affect its acceptability, particularly for preventive interventions, in which costs are incurred in the short term and the benefits are in the future. Most stakeholders prefer to delay undesirable effects and costs, rather than incur them in the present, and they value immediate desirable effects more than those in the future [44]. The size of this preference – the discount rate – varies, and some stakeholders might have a substantially higher or lower discount rate than others. In particular, discount rates may affect politicians’ decisions.

Disagreement amongst stakeholders regarding the values assigned to the desirable and undesirable effects of an option might also affect acceptability to some stakeholders. This might be due to their perceptions of the relative importance of the effects for others. Ethical principles, such as autonomy, non-maleficence, beneficence and justice [45, 46], may affect acceptability. For example, stakeholders for whom autonomy is especially important might be opposed to options that limit people’s autonomy, such as legislation that mandates the use of helmets or vaccines, even when they agree about the balance between the desirable and undesirable effects of those interventions.

In the example in Additional file 2, there was high certainty evidence that women readily engage with interventions designed to increase communication with and support from other pregnant women and healthcare providers, and that participatory women’s groups are likely to do this [47]. There was also high certainty evidence that healthcare providers are willing to supply pregnancy-related information and offer psychological and emotional support to women (either via women’s groups or antenatal visits) provided the resources and organisational support are available. Based on this evidence, the judgment of the panel was that women’s groups using PLA cycles are probably acceptable to all stakeholders.

#### Feasibility

Limitations in the feasibility of a health system or public health option can affect decisions about whether to recommend or implement the option. They can also have implications for how to implement the option, if it is recommended or implemented. It is unhelpful to recommend options that are impractical. Therefore, it is incumbent on health system and public health decision-makers to consider important barriers to implementing options and strategies to address any barriers they identify [48, 49].

In the example in Additional file 2, there was high certainty evidence that healthcare providers may need additional training if they are to be involved in facilitating women’s groups and this may be a barrier in some resource-poor settings. Women’s groups are also likely to require a reorganisation of services, and using existing public sector health workers and local health systems may be more feasible and more likely to succeed than project-based interventions [50]. Despite these barriers, the panel’s judgment was that the implementation of participatory women’s groups is probably feasible.

### Drawing conclusions

After making a judgment in relation to each criterion, panels must make a decision based on all of those judgments. Often, it will be straightforward for a panel to consider the implications of their judgments, and to make a decision; sometimes, it is not. Panels making health system or public health recommendations should make explicit assessments about the strength of their recommendations based on their judgments for all the criteria [5–7].

By strength of recommendations we mean the extent to which the panel can be confident, after considering all the relevant criteria, that all of the desirable consequences of an option outweigh all of its undesirable consequences (Additional file 1). A strong recommendation means the panel is confident that the desirable consequences outweigh the undesirable, or vice versa; a conditional recommendation means the panel is less confident. When panels make a conditional recommendation, they should provide clear guidance regarding the specific conditions that favour implementing or rejecting the option.

For all decisions, panels should provide a justification in which they summarise their judgments for the criteria that were most important for their decision. For difficult decisions or decisions on which panel members disagree, it can be helpful to consider explicitly the implication of each judgment and the weight given to each of the criteria.

In the example in Additional file 2, the panel made a conditional recommendation for women’s groups using PLA cycles in rural settings with low access to health services. Their justification for this decision was not provided in the guideline, but is likely to have been because the balance between the desirable and undesirable effects, the impact on equity, and the cost effectivity probably favour the option. The conditionality of recommending it in rural settings is likely because this is where the studies were performed, and the panel was uncertain about whether the effects would be the same in urban areas [19].

Policy-makers and managers making decisions (rather than recommendations) cannot make strong or conditional decisions. The choices available to them are fully implementing the option, implementing it and conducting an impact evaluation, conducting a pilot study prior to fully implementing it, implementing it only in some settings or populations, postponing a decision, or not implementing the option [51].

#### Subgroup considerations

Health system or public health decision-makers should reach conclusions about relevant subgroups or contexts, and include any important considerations in their conclusions. A previous WHO recommendation on community mobilisation through facilitated PLA cycles with women’s groups for maternal and newborn health [19] was used to inform the recommendation in Additional file 2. The panel for that WHO recommendation concluded that it is possible that PLA cycles with women’s groups could have different effects in urban versus rural areas, but elected not to make the recommendation for rural areas only because urban areas can also experience poor access and marginalisation. However, it also recommended that more research should be carried out in urban areas.

#### Implementation

Health system and public health options are often complex, and decision-makers may require detailed guidance on implementation. Key implementation considerations can typically be drawn from the evidence, additional considerations and judgments that were made regarding equity, acceptability and feasibility. Key organisational, political, social and resource considerations are listed in the framework in Additional file 2 for women’s groups using PLA cycles.

#### Monitoring and evaluation

Because there is often important uncertainty about the effects of health system and public health interventions, monitoring and evaluation considerations are a key element of most conclusions.

Decision-makers should consider what monitoring and evaluation will be necessary. They should consider what indicators are important to monitor, and whether impact evaluation is needed [51, 52]. When there is a lack of evidence or there are important uncertainties about the impacts of health system and public health interventions, it is particularly important to monitor and evaluate their implementation.

The panel in the participatory women’s groups example stipulated that, to ensure high quality implementation adapted to the local context, ongoing monitoring and evaluation is necessary. They also listed research priorities (Additional file 2).

#### Dissemination of EtD frameworks

The target audiences of health system and public health decisions may be policy-makers and managers deciding whether to implement policies, programmes or recommendations in their jurisdictions, or the people who are affected by those decisions.

People making policy decisions should be accountable to the people affected by their decisions. The EtD framework facilitates a transparent approach for policy-making and for documenting the basis for decisions so that this is accessible. Use of an interactive EtD tool makes it possible to generate presentations for different audiences, including a recommendation to decision presentation for policy-makers and managers considering whether to implement a recommendation in their jurisdiction. An example of a recommendation to decision presentation generated from the EtD framework in Additional file 2 can be found here. This presentation provides a structure for policy-makers and managers to discuss and decide about the adoption and adaptation of a recommendation. They can consider the same criteria as the panel who made the recommendation, while incorporating evidence specific for their setting and making judgments that are appropriate for their context. This is particularly important for global recommendations.

## Discussion

The health system and public health EtD framework supports systematic, structured and transparent use of evidence for recommendations and decisions. Policy-makers are likely to understand the summarised evidence presented in the framework better than systematic reviews [53], and the framework strengthens the credibility of decisions by documenting the evidence-based decision-making process. This includes showing how judgments were made when there was a lack of evidence.

The use of EtD frameworks requires ensuring that panel members are familiar with, and have a shared understanding of, the contents of the framework and its role in making decisions. This is a potential limitation of EtD frameworks. However, once this is achieved, panel members have reported that the frameworks help to structure discussion, often saving time, and ensure systematic and explicit consideration of all of the relevant factors [14].

Many governments and organisations responsible for health system and public health decisions do not have sufficient resources and expertise to prepare an optimal EtD framework. This is not a limitation of the EtD framework, but a practical limitation to whether the EtD framework is used or not. It will often be necessary to take shortcuts. In these situations, the EtD framework can still help decision-makers ensure that they use a systematic and transparent approach to making decisions, while enabling documentation of the evidence that  was used to inform their judgments, and how judgments were made when evidence was lacking. Indeed, as noted at the beginning of this article, key purposes of the EtD framework are to support panels by helping them to structure and document discussion, and to identify reasons for disagreements. The framework can help to improve decision-making regardless of the resources available to populate a framework with evidence or the availability of reliable evidence to inform decisions.

Using the EtD framework may require additional resources to synthesise evidence in addition to that of effectiveness, for example, qualitative evidence of acceptability and feasibility. This is sometimes critical, but it is not always necessary. For example, systematic reviews of qualitative evidence have been essential for some health system recommendations [20], but may not be needed if, for example, there is high certainty evidence of effects and large net benefits or harms, with little concern about the acceptability or feasibility of an intervention.

A strength of the EtD framework is its flexibility. In addition to flexibility with regards to how much effort goes into populating a framework with evidence, it is flexible in terms of the relative importance attached to the included criteria, since this depends on the nature of the decision being made. Moreover, the EtD framework can be adapted by organisations, for example, by modifying the key criteria that are included [23]. The interactive EtD tool [54] and GRADE’s official software GRADEpro GDT [55] include 16 templates for different types of recommendations and decisions for either two or multiple options. The templates include clinical recommendations from different perspectives, coverage decisions, recommendations about tests, and the four templates for health systems and public health recommendations and decisions shown in Box 1. It is possible for organisations to translate and adapt these to meet their specific mandate and needs.

WHO and other organisations that make health system and public health recommendations can facilitate the use of the EtD framework by policy-makers and managers by providing them with recommendation to decision presentations (such as the one in Additional file 3), which mirror the EtD frameworks used by panels making recommendations. Recommendations that use EtD frameworks can, in this way, substantially reduce the amount of work required by governments or organisations at national and sub-national levels and facilitate adaptation of recommendations to specific settings.

## Conclusions

The health system and public health EtD framework provides a structured and transparent approach to support policy-making informed by the best available research evidence, while making the basis for decisions accessible to those whom the decisions will affect. This framework can also be used to facilitate dissemination of recommendations, and enable decision-makers to adopt, and adapt, recommendations or decisions.

## Additional files

Additional file 1: Evidence to Decision Frameworks: Terminology. (DOCX 32 kb)

Additional file 2: Evidence to Decision Framework for women’s groups. (DOCX 332 kb)

Additional file 3: GRADE Recommendation to decision (RtD) presentation of an evidence to decision (EtD) framework for a health system and public health decision. (DOCX 29 kb)

## Abbreviations

EtD: Evidence to Decision
PLA: Participatory learning and action
