# Supplementary material for: The GRADE Evidence to Decision (EtD) framework for health system and public health decisions
Source: Health Res Policy Syst. 2018 May 29;16:45. doi: 10.1186/s12961-018-0320-2 (PMC5975536; doi:10.1186/s12961-018-0320-2)
Supplement: Supplementary file 1 — Evidence to Decision Frameworks: Terminology. (DOCX 32 kb) [file 12961_2018_320_MOESM1_ESM.docx]

**Additional file 1. Evidence to decision frameworks: Terminology**

| Certainty of the evidence (also called quality of evidence, or confidence in estimates effect) [^[[1]](#endnote-1)^,^[[2]](#endnote-2)^] | In the context of a systematic review, the ratings of the certainty of the evidence reflect the extent of our confidence that the estimates of the effect are correct. In the context of making decisions, the certainty ratings reflect the extent of our confidence that the estimates of an effect are adequate to support a particular decision or recommendation.   \| Ratings \| Definitions \| \| --- \| --- \| \| **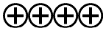**  High certainty \| The panel is very confident that the true effect lies close to that of the estimate of the effect \| \| **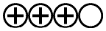**  Moderate certainty \| The panel is moderately confident in the effect estimate: The true effect is likely to be close to the estimate of the effect, but there is a possibility that it is substantially different \| \| 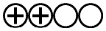  Low certainty \| The panel’s confidence in the effect estimate is limited: The true effect may be substantially different from the estimate of the effect \| \| **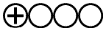**  Very low certainty \| The panel has very little confidence in the effect estimate: The true effect is likely to be substantially different from the estimate of effect \| |
| --- | --- | --- | --- | --- | --- | --- | --- | --- | --- | --- | --- |
| Decision-makers | We use the term ‘decision-makers’ to refer to individuals, or groups who make decisions about implementing or paying for an intervention. Their decisions may be based on recommendations. Decision-makers include clinicians, patients and the public, health policymakers and managers. |
| Decisions and recommendations | We use the term ‘decisions’ broadly to refer to both to decisions about recommendations (clinical practice guidelines and health system or public health recommendations) and to decisions about what to do (clinical decisions, coverage decisions, and policy or management decisions). We sometimes use the two terms together to be clear that we are referring to both types of decisions. EtD frameworks can be used for making recommendations (e.g. by guideline panels) or decisions (e.g. by policymakers). |
| Indicator [^[[3]](#endnote-3)^] | A quantitative or qualitative factor or variable that provides a simple and reliable means to measure achievement, to reflect the changes connected to an intervention, or to help assess performance |
| Intervention or option | For clinical recommendations and coverage decisions, we generally use intervention (and comparison) to refer to the alternatives compared in an EtD framework. For health system and public health recommendations or decisions, we generally use option (and alternative). In EtD frameworks for multiple (more than 2) alternatives, we use options. |
| Panel | A group of people making recommendations or decisions |
| Strength of recommendations [^[[4]](#endnote-4)^,^[[5]](#endnote-5)^] | The extent to which one can be confident that the desirable consequences of an intervention outweigh its undesirable consequences   \| Strong recommendation \| The panel is highly confident of the balance between desirable and undesirable consequences \| \| --- \| --- \| \| Weak (conditional, discretional, or qualified) recommendation \| The panel is less confident of the balance between desirable and undesirable consequences. We use the term ‘weak’ in this series, but some people prefer different terms and sometimes the context of a recommendation necessitates using a different term. \| |

1. Guyatt GH, Oxman AD, Kunz R, Vist GE, Falck-Ytter Y, Schunemann HJ, and the GRADE Working Group. What is ‘quality of evidence’ and why is it important to clinicians? BMJ 2008; 336:995-8. [↑](#endnote-ref-1)
2. Balshem H, Helfand M, Schunemann H, Oxman AD, Kunz R, Brozek J, et al. GRADE guidelines 3. Rating the quality of evidence – introduction. J Clin Epidemiol 2011; 64:401-6. [↑](#endnote-ref-2)
3. Fretheim A, Oxman AD, Lavis JN, Lewin S. SUPPORT Tools for evidence-informed health Policymaking (STP). 18. Planning monitoring and evaluation of policies. Health Res Policy Syst. 2009, 7(Suppl 1):S18. [↑](#endnote-ref-3)
4. Guyatt GH, Oxman AD, Kunz R, Falck-Ytter Y, Vist GE, Liberati A, Schunemann HJ, and the GRADE Working Group. Going from evidence to recommendations. BMJ 2008; 336:1049-51. [↑](#endnote-ref-4)
5. Andrews J, Guyatt GH, Oxman AD, Alderson P, Dahm P, Falck-Ytter Y, et al. GRADE guidelines: 14. Going from evidence to recommendations: the significance and presentation of recommendations. J Clin Epidemiol 2013 Jan 9. pii: S0895-4356(12)00138-2. doi: 10.1016/j.jclinepi.2012.03.013. [↑](#endnote-ref-5)
