# Supplementary material for: The GRADE Evidence to Decision (EtD) framework for health system and public health decisions
Source: Health Res Policy Syst. 2018 May 29;16:45. doi: 10.1186/s12961-018-0320-2 (PMC5975536; doi:10.1186/s12961-018-0320-2)
Supplement: Supplementary file 2 — Evidence to Decision Framework for women’s groups. (DOCX 332 kb) [file 12961_2018_320_MOESM2_ESM.docx]

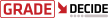
 *Interactive Evidence to Decision Framework*

*Pablo Alonso-Coello, Jenny Moberg, and Andy Oxman prepared this Evidence to Decision framework based on two WHO guidelines: one on antenatal care for a positive pregnancy experience*[WHO 2016] *and one on* *community mobilization through facilitated participatory learning and action cycles with women's groups for maternal and newborn health*[WHO 2014]*- 24 February 2017.*

An interactive version of this framework is available [here](https://ietd.epistemonikos.org/#/frameworks/585a48c30757406e13b8193c/question).

**Should women’s groups using participatory learning and action (PLA) cycles be used to achieve a positive pregnancy experience?**

(Health system and public health recommendation)

QUESTION

***Question details***

**Problem:** Achieving a positive pregnancy experience

**Option:** Women’s groups using participatory learning and action (PLA) cycles

**Comparison:** Usual care

**Main outcomes:**

- Four or more antenatal care visits
- One or more antenatal care visits
- Delivery in a health facility
- Perinatal deaths
- Maternal deaths

**Setting:** Global

**Perspective:** Population

-----------------------------------------------------------------------------------------------------------------------------

***Background***

Antenatal care (ANC) uptake is low in many regions of the world, with only 64% of pregnant women receiving the recommended minimum of four ANC visits [WHO 2016]. Increasing the dialogue around barriers and facilitators to utilizing antenatal care services and keeping healthy during pregnancy, and providing women and their partners with support in addressing challenges they may face, may lead to improved ANC uptake and a positive pregnancy experience.

This framework presents the research evidence and other relevant information on women’s groups using participatory learning and action (PLA) cycles to achieve a positive pregnancy experience. **Women’s groups using PLA cycles** are facilitated by trained facilitators, with the aim of identifying, prioritising and addressing problems women face around pregnancy, childbirth and after birth, and empowering women to seek care and choose healthy pregnancy and newborn care behaviours. Meetings are usually held on a monthly basis and activities are differentially prioritized according to the local context and conditions.

**A positive pregnancy experience** is defined as maintaining physical and sociocultural normality, maintaining a healthy pregnancy for mother and baby (including preventing or treating risks, illness and death), having an effective transition to positive labour and birth, and achieving positive motherhood (including maternal self-esteem, competence and autonomy).

ASSESSMENT

***Problem***

Is the problem a priority?

**Judgment**

| Don't know | Varies | No | Probably No | Probably Yes | Yes |
| --- | --- | --- | --- | --- | --- |

**Research evidence**

Approximately 303 000 women and adolescent girls died as a result of pregnancy and childbirth-related complications in 2015 [Alkema 2016]. Around 99% of maternal deaths occur in low-resource settings and most can be prevented [WHO 2014a]. Similarly, approximately 2.6 million babies were stillborn in 2015, also mainly in low-resource settings [Blencowe 2016]. Nevertheless, there is evidence that effective interventions exist at reasonable cost for the prevention or treatment of virtually all life-threatening maternal complications [Campbell 2006], and almost two thirds of the global maternal and neonatal disease burden could be alleviated through optimal adaptation and uptake of existing research findings [Fisk 2011].

**Additional considerations**

None

***Desirable effects***

How substantial are the desirable anticipated effects?

**Judgment**

| Don't know | Varies | Trivial | Small | Moderate | Large |
| --- | --- | --- | --- | --- | --- |

**Research evidence**

Summary of findings: [(See an interactive version here)](http://isof.epistemonikos.org/#finding/)


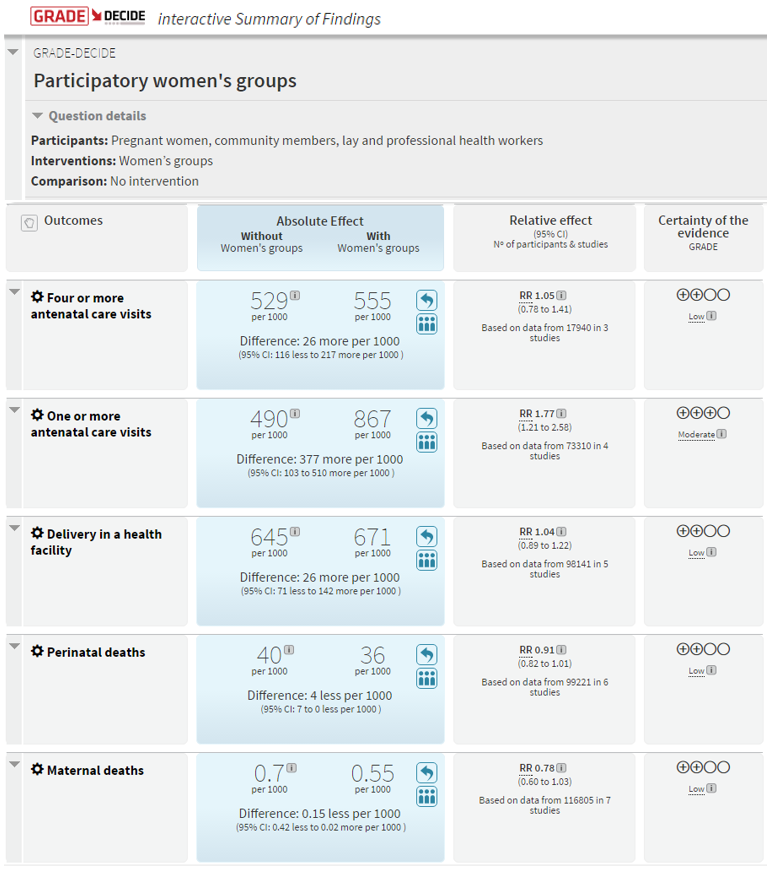


**Additional considerations**

Seven cluster-randomized trials conducted between 1999 and 2011 contributed data to this comparison. Trials were conducted in Bangladesh (2), India (2), Malawi (2), and Nepal (1), and six out of seven were conducted in rural settings. Most of the participants were women aged 15 to 49 years. The intervention comprised involving women in participatory learning and action cycles, facilitated by trained facilitators, with the aim of identifying, prioritising and addressing problems women face around pregnancy, childbirth and after birth; and empowering women to seek care and choose healthy pregnancy and newborn care behaviours [Prost 2013]. Meetings were usually held on a monthly basis and activities were differentially prioritized according to the local context and conditions.

Coverage of women’s group meetings ranged from one group per 309 to 1414 people in the included trials, with the percentage of pregnant women attending groups ranging from 2% to 51%. Five out of seven trials were conducted against a backdrop of context-specific health system strengthening in both intervention and control arms; these included training of traditional birth attendants (TBAs) and provision of basic equipment to TBAs or primary care facilities in four trials. Sensitivity analyses were performed by excluding three trials with low coverage (less than 30% of women’s groups comprising pregnant women).

The pathways of influence of this multifaceted, context-specific intervention on maternal and newborn outcomes are difficult to assess. Communication and support probably play an important role, but other mechanisms may also be operating.

Findings from the review conducted for this guideline are consistent with a 2013 review of women’s groups using PLA cycles [Prost 2013] which formed the evidence base for the 2014 WHO recommendation on community mobilization through facilitated participatory learning and action cycles with women’s groups [WHO 2014]. That recommendation is as follows: “The implementation of community mobilization through facilitated participatory learning and action cycles with women’s groups is recommended to improve maternal and newborn health, particularly in rural settings with low access to health services (strong recommendation; moderate-quality evidence on neonatal mortality, low-quality evidence for maternal mortality and care-seeking outcomes).”

***Undesirable effects***

How substantial are the undesirable anticipated effects?

**Judgment**

| Don't know | Varies | Large | Moderate | Small | Trivial |
| --- | --- | --- | --- | --- | --- |

**Research evidence**

See iSoF table above. No adverse effects of women’s groups were identified.

**Additional considerations**

See Additional considerations above. No adverse effects of women’s groups were identified.

***Certainty of the evidence***

What is the overall certainty of the evidence of effects?

**Judgment**

| No included studies | Very low | Low | Moderate | High |
| --- | --- | --- | --- | --- |

**Research evidence**

See iSoF table above.

**Additional considerations**

See Additional considerations above.

***Values***

Is there important uncertainty about, or variability in, how much people value the main outcomes?

**Judgment**

| Important uncertainty or variability | Possibly important uncertainty or variability | Probably no important uncertainty or variability | No important uncertainty or variability |
| --- | --- | --- | --- |

**Research evidence**

A scoping review of what women want from ANC and what outcomes they value informed the ANC guideline [Downe 2016a]. Evidence showed that women from high-, medium- and low-resource settings valued having a positive pregnancy experience, and that they value the opportunity to communicate with, and receive support from, other pregnant women and healthcare providers, as an integral part of that positive experience (high confidence in the evidence).^1^

**Additional considerations**

The systematic review of effects found moderate, but uncertain benefits of women’s groups [Mbuagbaw 2015]. In addition, women’s groups very likely increase communication and social support, which women value. Given that no adverse effects were identified, it is unlikely that there is important uncertainty or variability in how much women value the main outcomes.

***Balance of effects***

Does the balance between desirable and undesirable effects favour the option or the comparison?

**Judgment**

| Don't know | Varies | Favours the comparison | Probably favours the comparison | Does not favour either the option or the comparison | Probably favours the option | Favours the option |
| --- | --- | --- | --- | --- | --- | --- |

**Research evidence**

See the four preceding criteria.

**Additional considerations**

See the four preceding criteria.

***Resources required***

How large are the resource requirements (costs)?

**Judgment**

| Don't know | Varies | Large costs | Moderate costs | Negligible costs or savings | Moderate savings | Large savings |
| --- | --- | --- | --- | --- | --- | --- |

**Research evidence**

Costs of these interventions are difficult to estimate and depend on context [Mangham-Jefferies 2014]. Costing must take into account the facilitators’ time, training and supervision; these elements are considered key to the quality of implementation and the success of the intervention. Estimated costs (in 2011 international dollars) for participatory women’s group interventions based on [Prost 2013] are summarised in the table below.

|  | Cost per neonatal death averted | Cost per 100 births |
| --- | --- | --- |
| Nepal | $22,961 | $24,245 |
| India | $2,770 | $4,431 |
| Malawi | $17,604 | $19,521 |
| Bangladesh | $19,810 | $20,530 |

**Additional considerations**

None

***Certainty of evidence of required resources***

What is the certainty of the evidence of resource requirements (costs)?

**Judgment**

| No included studies | Very low | Low | Moderate | High |
| --- | --- | --- | --- | --- |

**Research evidence**

There is variability in the costs reported across studies.

**Additional considerations**

Costs are likely to vary across countries and may vary across settings within countries.

***Cost-effectiveness***

Does the cost-effectiveness of the option favour the option or the comparison?

**Judgment**

| Don't know | Varies | Favours the comparison | Probably favours the comparison | Does not favour either the option or the comparison | Probably favours the option | Favours the option |
| --- | --- | --- | --- | --- | --- | --- |

**Research evidence**

A systematic review of the cost–effectiveness of strategies to improve the utilization and provision of maternal and new-born health care in low- and lower-middle-income countries reported that there was reasonably strong evidence for the cost–effectiveness of women’s groups using PLA cycles [Mangham-Jefferies 2014]. Estimated costs per life saved for these interventions was US$ 268. Although the incremental cost per neonatal death averted differed between trials, women’s groups practicing participatory learning and action were a highly cost-effective intervention in these trials, relative to WHO-recommended standards [Prost 2013].

**Additional considerations**

None

***Equity***

What would be the impact on health equity?

**Judgment**

| Don't know | Varies | Reduced | Probably reduced | Probably no impact | Probably increased | Increased |
| --- | --- | --- | --- | --- | --- | --- |

**Research evidence**

One of the trials included in the review evaluated the impact on equity of women’s groups [Houweling 2013, Tripathy 2010]. Findings indicated that the effect of participatory women’s groups was greatest among the most socio-economically marginalized, with neonatal mortality rates 59% lower in the intervention group than control group, compared with 36% lower in the less marginalized group.

**Additional considerations**

Interventions such as women’s groups using PLA cycles, community mobilization, and home visits during pregnancy are a way of facilitating dialogue and action with, and empowering, disadvantaged populations to engage in efforts to improve health and to strengthen broader community support. Women’s groups using PLA cycles were conducted in marginalized areas where other support mechanisms often do not exist.

In low- and middle-income countries, women who are poor, least educated, and residing in rural areas have lower health intervention coverage and worse pregnancy outcomes than the more advantaged women (WHO 2015). Less-educated women are more likely to have lower maternal health literacy; therefore, effective interventions that improve maternal health literacy should reduce health inequalities by improving women’s ability to access, understand, and use educational materials [Lori 2016].

In addition, emotional, psychological, cultural and social support is often lacking for disadvantaged women and in low resourced settings. Improving support for women during pregnancy is likely to help to facilitate learning, engagement with care, and a positive pregnancy experience.  Whilst access to comprehensive information around pregnancy is a basic human right, disadvantaged women often have to travel long distances and wait long hours to be seen, with limited opportunity for effective dialogue during their visits with health workers. Information in the form of brochures, videos, or case-notes may not be in an educationally or culturally accessible format. Due to these and other barriers, such as poor perceived quality of care, disadvantaged women have less access to pregnancy-related information, and less opportunity to communicate their needs. Therefore, interventions such as participatory women’s groups are a way of facilitating dialogue with, and empowering, disadvantaged populations to engage with healthcare promotion, and to access support.

***Acceptability***

Is the option acceptable to key stakeholders?

**Judgment**

| Don't know | Varies | No | Probably No | Probably Yes | Yes |
| --- | --- | --- | --- | --- | --- |

**Research evidence**

Evidence from a qualitative systematic review [Downe S 2016b] suggests that women in a variety of settings and contexts readily engage with interventions designed to increase communication and support provided they are delivered in a caring and respectful manner (high confidence in the evidence).^1^ The use of women’s groups is likely to fulfil two key requirements of ANC from a woman’s perspective – the opportunity to receive and share relevant information and the opportunity to develop supportive relationships with other pregnant women and healthcare providers (high confidence in the evidence).^1^

Women and providers in LMICs also highlighted the importance of active community engagement in the design and delivery of informational based services especially in communities where traditional beliefs may differ from conventional understandings (moderate confidence in the evidence).^1^

Evidence from providers in a parallel systematic review suggests that there is a willingness to supply pregnancy related information and offer psychological/emotional support to women (either via women’s groups or antenatal visits) provided the resources are available (high confidence in the evidence)^1^ and the services are delivered in a co-ordinated, organized manner with appropriate managerial support (moderate confidence in the evidence)^1^ [Downe 2016c].

**Additional considerations**

None

***Feasibility***

Is the option feasible to implement?

**Judgment**

| Don't know | Varies | No | Probably No | Probably Yes | Yes |
| --- | --- | --- | --- | --- | --- |

**Research evidence**

Qualitative evidence suggests that, where health-care providers are involved in facilitating women’s groups, they may need additional training to help with the facilitative components and this may be a barrier in some resource-poor settings (high confidence in the evidence)^1^ [Downe 2016c].

Women’s groups are likely to require a reorganization of services, and evidence from a variety of settings suggests that a clear, positive and inclusive managerial style would be required to implement changes of this nature (moderate confidence in the evidence)^1^ [Downe 2016c].

**Additional considerations**

Community-based interventions introduced through existing public sector health workers and local health systems may be more feasible and more likely to succeed than project based interventions [Memon 2015].

CONCLUSIONS

***Summary of judgments***

| Problem | -  Don't know | -  Varies |  | -  No | -  Probably No | -  Probably Yes | **✓**  Yes |
| --- | --- | --- | --- | --- | --- | --- | --- |
| Desirable effects | -  Don't know | -  Varies |  | -  Trivial | -  Small | **✓**  Moderate | -  Large |
| Undesirable effects | -  Don't know | -  Varies |  | -  Large | -  Moderate | -  Small | **✓**  Trivial |
| Certainty of the evidence | -  No included studies |  |  | -  Very low | **✓**  Low | -  Moderate | -  High |
| Values |  |  |  | -  Important uncertainty or variability | -  Possibly important uncertainty or variability | **✓**  Probably no important uncertainty or variability | -  No important uncertainty or variability |
| Balance of effects | -  Don't know | -  Varies | -  Favours the comparison | -  Probably favours the comparison | -  Does not favour either the option or the comparison | **✓**  Probably favours the option | -  Favours the option |
| Resources required | -  Don't know | -  Varies | -  Large costs | **✓**  Moderate costs | -  Negligible costs or savings | -  Moderate savings | -  Large savings |
| Certainty of evidence of required resources | -  No included studies |  |  | -  Very low | **✓**  Low | -  Moderate | -  High |
| Cost-effectiveness | -  Don't know | -  Varies | -  Favours the comparison | -  Probably favours the comparison | -  Does not favour either the option or the comparison | **✓**  Probably favours the option | -  Favours the option |
| Equity | -  Don't know | -  Varies | -  Reduced | -  Probably reduced | -  Probably no impact | **✓**  Probably increased | -  Increased |
| Acceptability | -  Don't know | -  Varies |  | -  No | -  Probably No | **✓**  Probably Yes | -  Yes |
| Feasibility | -  Don't know | -  Varies |  | -  No | -  Probably No | **✓**  Probably Yes | -  Yes |

***Type of recommendation***

**Judgment**

| Strong recommendation against the option | Conditional recommendation against the option | Conditional recommendation for either the option or the comparison | Conditional recommendation for the option | Strong recommendation for the option |
| --- | --- | --- | --- | --- |

***Recommendation***

*Recommendation*

The implementation of community mobilization through women's groups using facilitated participatory learning and action (PLA) cycles is recommended to improve maternal and newborn health, particularly in rural settings with low access to health services. Participatory women’s groups represent an opportunity for women to discuss their needs during pregnancy and the barriers to reaching care, and to increase communication and support to pregnant women.

Detailed information and guidance related to the recommendation, including important implementation considerations, can be found in the 2014 WHO recommendation on women’s groups using PLA cycles [WHO_2014].

***Justification***

Women’s groups using PLA cycles may reduce the number of perinatal deaths and increase the number of women who have one or more antenatal care visits. Moreover, they provide an opportunity for communication and support, which women value, and they probably increase equity. They also empower disadvantaged populations to engage in efforts to improve health. They are probably cost-effective, although the costs of implementing women’s groups using PLA cycles vary.

**Detailed justification**

| Problem Desirable effects Undesirable effects Certainty of the evidence Values Balance of effects  Resources required Certainty of evidence of required resources Cost-effectiveness Equity  Acceptability Feasibility | The certainty of the evidence is low.  Women’s groups using PLA cycles may reduce the number of perinatal deaths, increase the number of women who have one or more antenatal care visits, and lead to a positive pregnancy experience.  Women’s groups using PLA cycles are probably cost-effective.  Women’s groups using PLA cycles probably increase equity by benefiting underserved populations, and they empower disadvantaged populations. |
| --- | --- |

***Subgroup considerations***

Not applicable.

***Implementation considerations***

Based on [WHO 2014]

- To have an impact on health outcomes, the time period of the intervention should be no shorter than three years.
- There needs to be adequate coverage of the intervention in terms of density of groups in the population. There is some evidence that women’s groups might be more successful where more than 30% of pregnant women participate, however the evidence at present is not definitive. The effect may also vary by context. For example, it may depend on prior existence, strength and cohesion of local social networks.
- High quality facilitators are key in establishing and maintaining groups and helping them to be effective. Good training and support of facilitators are therefore essential.
- Although it is a ‘community intervention’, like any intervention at large scale, it must be supported by appropriate structures, systems and processes. For example, each facilitator should be responsible for no more than 8-10 groups per month to act effectively, and resources must be in place to support this.
- Implementation should include awareness of potential harms (e.g. gender violence, conflict with health providers or other community members). Potential harms should be monitored throughout implementation so that they can be managed.

**The political/social context**

- Political support (national and local level) is essential.
- The intervention must be adapted to reflect each country’s context, specific capacities and constraints.
- Implementing the intervention as part of national community health developmental strategies/plans or other community development structures is likely to enhance coverage and sustainability.
- The women’s groups should not operate in isolation. To be effective they need the cooperation of other social groups, and responsive and accountable health services. Co-operation from non-health sectors may be crucial for implementing group plans; e.g. road maintenance.

**Specific local factors that might be relevant to implementation**

- History of participation in communities, existence of other groups, local decision making structures and processes should be taken into account in design/implementation.
- Data are needed on local barriers and facilitators of implementation, and on acceptability of the intervention to women.
- Implementation should consider the role of men and other members of the community (e.g. religious groups, mothers-in-law) and how and when they participate in the process.
- The design of the process used with groups should be adapted according to the groups in question; e.g. accounting for levels of literacy and numeracy, preferences for oral versus visual methods.
- Ethnic group mix, religion, caste and other social categories affecting group dynamics need to be considered in developing the approach; e.g. how and where groups are formed.

***Monitoring and evaluation***

- Implementation of this recommendation should be subject to ongoing monitoring and evaluation to ensure high quality implementation adapted to the local context.

***Research priorities***

- Determine what types of participatory women’s group models are best, including the optimum group size, the type of participants (pregnant women only, women of reproductive age, all women), type and number of facilitators, and content of health promotion.
- Determine the feasibility and acceptability of mixed gender groups.
- Determine the best model for integration/cooperation with the facility-based/local health system.
- Determine the optimal models for community mobilisation.
- Explore the mechanism of effects behind this option.
- More research is needed to understand the effects of community mobilization on care-seeking outcomes in different contexts.

EVIDENCE PROFILE

World Health Organization. [Community-based interventions to improve communication and support](http://apps.who.int/iris/bitstream/10665/250796/8/9789241549912-websupplement-eng.pdf?ua=1). In: WHO recommendations on antenatal care for a positive pregnancy experience: evidence base. Geneva: World Health Organization, 2016; 108-10.

REFERENCES

[Alkema 2016] Alkema L, Chou D, Hogan D, Zhang S, Moller A-B, Gemmill A, et al. [United Nations Maternal Mortality Estimation Inter-Agency Group collaborators and technical advisory group. Global, regional, and national levels and trends in maternal mortality between 1990 and 2015, with scenario-based projections to 2030: a systematic analysis by the UN Maternal Mortality Estimation Inter-Agency Group](http://www.thelancet.com/journals/lancet/article/PIIS0140-6736(15)00838-7/abstract). Lancet. 2016 Jan 30;387(10017):462-74. doi: 10.1016/S0140-6736(15)00838-7.

[Blencowe 2016] Blencowe H, Cousens S, Bianchi Jassir F, Chou D, Mathers C, et al. [National, regional, and worldwide estimates of stillbirth rates in 2015, with trends from 2000: a systematic analysis](http://www.thelancet.com/journals/langlo/article/PIIS2214-109X(15)00275-2/abstract). Lancet Glob Health. 2016 Feb;4(2):e98-e108. doi: 10.1016/S2214-109X(15)00275-2.

[Campbell 2006] Campbell OMR, Graham WJ. [The Lancet Maternal Survival Series Steering Group. Strategies for reducing maternal mortality: getting on with what works](http://www.thelancet.com/journals/lancet/article/PIIS0140-6736(06)69381-1/abstract). Lancet. 2006 Oct 7;368(9543):1284-99. doi: 10.1016/S0140-6736(06)69381-1.

[Downe 2016a] Downe S, Finlayson K, Tunçalp Ӧ, Metin Gülmezoglu A. [What matters to women: a systematic scoping review to identify the processes and outcomes of antenatal care provision that are important to healthy pregnant women](http://onlinelibrary.wiley.com/doi/10.1111/1471-0528.13819/full). BJOG. 2016 Mar;123(4):529-39. doi: 10.1111/1471-0528.13819.

[Downe 2016b] Downe S, Finlayson K, Tunçalp Ö, Gülmezoglu AM. [Factors that influence the uptake of routine antenatal services by pregnant women: a qualitative evidence synthesis](http://onlinelibrary.wiley.com/doi/10.1002/14651858.CD012392/full). Cochrane Database Syst Rev. 2016 Oct 20;(10):CD012392. doi: 10.1002/14651858.CD012392.

[Downe 2016c] Downe S, Finlayson K, Tunçalp Ö, Gülmezoglu AM. Factors that influence the provision of good quality routine antenatal care services by health staff: a qualitative evidence synthesis. (unpublished).

[Fisk 2011] Fisk NM, McKee M, Atun R. [Relative and absolute addressability of global disease burden in maternal and perinatal health by investment in R&D](http://onlinelibrary.wiley.com/doi/10.1111/j.1365-3156.2011.02778.x/full). Trop Med Int Health. 2011 Jun;16(6):662-8. doi: 10.1111/j.1365-3156.2011.02778.x.

[Houweling 2013] Houweling TA, Tripathy P, Nair N, Rath S, Rath S, Gope R, et al. [The equity impact of participatory women's groups to reduce neonatal mortality in India: secondary analysis of a cluster-randomised trial](https://academic.oup.com/ije/article/42/2/520/736210/The-equity-impact-of-participatory-women-s-groups). Int J Epidemiol. 2013 Apr;42(2):520-32. doi: 10.1093/ije/dyt012.

[Lewin 2015] Lewin S, Glenton C, Munthe-Kaas H, Carlsen B, Colvin CJ, Gülmezoglu M, et al. [Using qualitative evidence in decision making for health and social interventions: an approach to assess confidence in findings from qualitative evidence syntheses (GRADE-CERQual)](http://journals.plos.org/plosmedicine/article?id=10.1371/journal.pmed.1001895). PLoS Med. 2015 Oct 27;12(10):e1001895. doi: 10.1371/journal.pmed.1001895.

[Lori 2016] Lori JR, Munro ML, Chuey MR. [Use of a facilitated discussion model for antenatal care to improve communication](http://www.sciencedirect.com/science/article/pii/S002074891500098X). Int J Nurs Stud. 2016 Feb;54:84-94. doi: 10.1016/j.ijnurstu.2015.03.018.

[Mangham-Jefferies 2014] Mangham-Jefferies L, Pitt C, Cousens S, Mills A, Schellenberg J. [Cost-effectiveness of strategies to improve the utilization and provision of maternal and newborn health care in low-income and lower-middle-income countries: a systematic review](https://bmcpregnancychildbirth.biomedcentral.com/articles/10.1186/1471-2393-14-243). BMC Pregnancy Childbirth. 2014 Jul 22;14:243. doi: 10.1186/1471-2393-14-243.

[Mbuagbaw 2015] Mbuagbaw L, Medley N, Darzi AJ, Richardson M, Habiba Garga K, Ongolo-Zogo P. [Health system and community level interventions for improving antenatal care coverage and health outcomes](http://onlinelibrary.wiley.com/doi/10.1002/14651858.CD010994.pub2/abstract). Cochrane Database Syst Rev. 2015 Dec 1;(12):CD010994. doi: 10.1002/14651858.CD010994.pub2.

[Memon 2015] Memon ZA, Khan GN, Soofi SB, Baig IY, Bhutta ZA. [Impact of a community-based perinatal and newborn preventive care package on perinatal and neonatal mortality in a remote mountainous district in Northern Pakistan](https://bmcpregnancychildbirth.biomedcentral.com/articles/10.1186/s12884-015-0538-8). BMC Pregnancy Childbirth. 2015;15:106. doi:10.1186/s12884-015-0538-8.

[Prost 2013] Prost A, Colbourn T, et al. [Women's groups practising participatory learning and action to improve maternal and newborn health in low-resource settings: a systematic review and meta-analysis](http://www.thelancet.com/journals/lancet/article/PIIS0140-6736(13)60685-6/abstract). Lancet. 2013 May 18;381(9879):1736-46. doi: 10.1016/S0140-6736(13)60685-6.

[Tripathy 2010] [Effect of a participatory intervention with women’s groups on birth outcomes and maternal depression in Jharkhand and Orissa, India: a cluster-randomised controlled trial](http://www.thelancet.com/journals/lancet/article/PIIS0140-6736(09)62042-0/abstract). Lancet. 2010 Apr 3;375(9721):1182-92. doi: 10.1016/S0140-6736(09)62042-0.

[WHO 2014] World Health Organization. [WHO recommendation on community mobilization through facilitated participatory learning and action cycles with women’s groups for maternal and newborn health](http://apps.who.int/iris/bitstream/10665/127939/1/9789241507271_eng.pdf?ua=1). Geneva: World Health Organization, 2014.

[WHO 2014a] World Health Organization. [Maternal mortality](http://www.who.int/mediacentre/factsheets/fs348/en/index.html). Fact sheet No. 348. Geneva: World Health Organization, 2014.

[WHO 2015] World Health Organization. [WHO recommendations on health promotion interventions for maternal and newborn health 2015](http://www.who.int/maternal_child_adolescent/documents/health-promotion-interventions/en/). Geneva: World Health Organization, 2015.

[WHO 2016] World Health Organization. [WHO recommendations on antenatal care for a positive pregnancy experience](http://www.who.int/reproductivehealth/publications/maternal_perinatal_health/anc-positive-pregnancy-experience/en/). Geneva: World Health Organization, 2016.

FOOTNOTES

1. Confidence in the evidence was assessed using the GRADE-CERQual approach [Lewin 2015].
