# Supplementary material for: The GRADE Evidence to Decision (EtD) framework for health system and public health decisions
Source: Health Res Policy Syst. 2018 May 29;16:45. doi: 10.1186/s12961-018-0320-2 (PMC5975536; doi:10.1186/s12961-018-0320-2)
Supplement: Supplementary file 3 — GRADE Recommendation to decision (RtD) presentation of an evidence to decision (EtD) framework for a health system and public health decision. (DOCX 29 kb) [file 12961_2018_320_MOESM3_ESM.docx]

**Additional file 3: GRADE Recommendation to decision (RtD) presentation of an evidence to decision (EtD) framework for a health system and public health decision**

A recommendation to decision (RtD) presentation of an evidence to decision (EtD) framework for a decision about women’s groups using participatory learning and action (PLA) cycles can be found here:

<https://ietd.epistemonikos.org/#/summary/58c57b6e07574006fb7614ef/policymakers>

The EtD framework from which this RtD presentation was generated was prepared by Pablo Alonso-Coello, Jenny Moberg, and Andy Oxman based on two WHO guidelines: one on antenatal care for a positive pregnancy experience [WHO 2016] and one on community mobilization through facilitated participatory learning and action cycles with women's groups for maternal and newborn health [WHO 2014] - 24 February 2017.

[WHO_2014] World Health Organization. [WHO recommendation on community mobilization through facilitated participatory learning and action cycles with women’s groups for maternal and newborn health](http://www.who.int/iris/bitstream/10665/127939/http:/apps.who.int/iris/bitstream/10665/127939/1/9789241507271_eng.pdf?ua=1). Geneva: World Health Organization, 2014.

[WHO_2016] World Health Organization. Community-based interventions to improve communication and support. In: [WHO recommendations on antenatal care for a positive pregnancy experience: evidence base.](http://www.who.int/reproductivehealth/publications/maternal_perinatal_health/anc-positive-pregnancy-experience/en/) Geneva: World Health Organization, 2016; 108-10.
